# Recent advances in understanding the role of metabolic heterogeneities in cell migration

**DOI:** 10.12703/r/10-8

**Published:** 2021-01-28

**Authors:** Jenna A Mosier, Yusheng Wu, Cynthia A Reinhart-King

**Affiliations:** 1 Department of Biomedical Engineering, Vanderbilt University, Nashville, TN, USA

**Keywords:** cancer, collective migration, ATP, heterogeneity, metabolism, extracellular matrix

## Abstract

Migration is an energy-intensive, multi-step process involving cell adhesion, protrusion, and detachment. Each of these steps require cells to generate and consume energy, regulating their morphological changes and force generation. Given the need for energy to move, cellular metabolism has emerged as a critical regulator of both single cell and collective migration. Recently, metabolic heterogeneity has been highlighted as a potential determinant of collective cell behavior, as individual cells may play distinct roles in collective migration. Several tools and techniques have been developed and adapted to study cellular energetics during migration including live-cell probes to characterize energy utilization and metabolic state and methodologies to sort cells based on their metabolic profile. Here, we review the recent advances in techniques, parsing the metabolic heterogeneities inherent in cell populations and their contributions to cell migration.

## Introduction

Cellular bioenergetics play a significant role in many essential biological processes including growth and proliferation, allowing cells to adapt to a changing environment^1^. Cellular energy utilization and metabolic plasticity can improve cell fitness and regulate disease progression by maximizing energy production and providing the necessary intermediates for biosynthetic processes^2–7^. However, it has only recently been reported that cell metabolism may play an important role in migration^8,9^, with intracellular energy generation still being explored.

Recent evidence suggests that cells have distinct energetic needs depending on their mode of migration^3^. Cells can move both as individual cells and as collective cohorts, adopting a variety of migratory modes, ranging from Rac1-dependent mesenchymal^10–13^ to RhoA-mediated amoeboid migration^14–16^. In addition to plasticly switching between these migratory modes or adopting intermediate characteristics of each^11,16–21^, migratory cells are also able to switch between two main metabolic pathways: glycolysis or oxidative phosphorylation (OXPHOS)^22^. Energy requirements depend on cell morphology, physical properties of surrounding environments, and cell–environment interactions and are therefore determining factors in cell migration^3^.

Collective migration is characteristic of a number of biological processes, including development, wound healing, and invasive diseases like cancer, where energy utilization and efficiency have recently been shown to play a significant role^23–26^. It has been reported that both single and collective cell migration may demand that certain energetic requirements be met during migration^9,27–30^. In single cells, the more energy-efficient, mitochondrial ATP generation occurs at the leading edge of cells^8^, whereas during collective migration, there is no consensus in the current literature on energy production at the leading edge of the cell front compared to the central follower cells^31,32^. Cell metabolic pathways and energy sources are of key interest in the context of cancer, as a better understanding of these processes could provide efficient targets for treating cancer and inhibiting cell invasion. Therefore, newly developed tools and techniques available for studying cell metabolism during migration have enabled further investigation of cellular energetic needs and the mechanisms by which energy is generated.

## Molecular probes for studying cell metabolism during migration

Cell metabolism has primarily been characterized using bulk techniques analyzing metabolic genes and proteins and with tools such as the Seahorse Analyzer XF that reports oxygen consumption and extracellular acidification rates of cell populations^33–36^. However, as more is learned on the heterogeneity of cell populations and migratory ability, it becomes clear that understanding individual cell energetics can shed light on these complex and intricate processes. Molecular probes that can be stably expressed in multiple cell lines are able to map these minute changes on a cell-by-cell basis to study single cell energetics in the context of larger cell populations. Many fluorescent biosensors thus exist to interrogate cell metabolism in real time, including those measuring the metabolic intermediates lactate^37^ and pyruvate^38^ or mitochondrial membrane potential^39^. Here, we have focused on just a few of the many probes used to investigate cell migration and metabolism.

### PercevalHR probe: visualizing ATP:ADP ratios

Adenosine triphosphate (ATP) and adenosine diphosphate (ADP) are two key components of energy transfer in cells, with ATP being essential in multiple migratory mechanisms including actomyosin contractility^40^, actin polymerization^41^, and cytoskeletal remodeling^42,43^. ATP:ADP ratios are useful in reporting the availability of these metabolic intermediates. The PercevalHR probe boasts the ability to track real-time ATP:ADP ratios and localization as cells migrate, providing substantial insight about the relationship between cell energy utilization and migration in various cell microenvironments^44^. PercevalHR combines a fluorescent protein with the bacterial regulatory protein GlnK1 that competitively binds to active ATP and ADP^44,45^. The probe has two distinct excitation wavelengths for ATP and ADP binding and has been successfully used to measure a ratio of these values in yeast cells^46^, HeLa cells^47^, pancreatic beta cells^48,49^, neuronal cells^50^, fibroblasts^51^, metastatic breast cancer cells^9,30^, and many others to report cell energy status.

**Figure 1.**
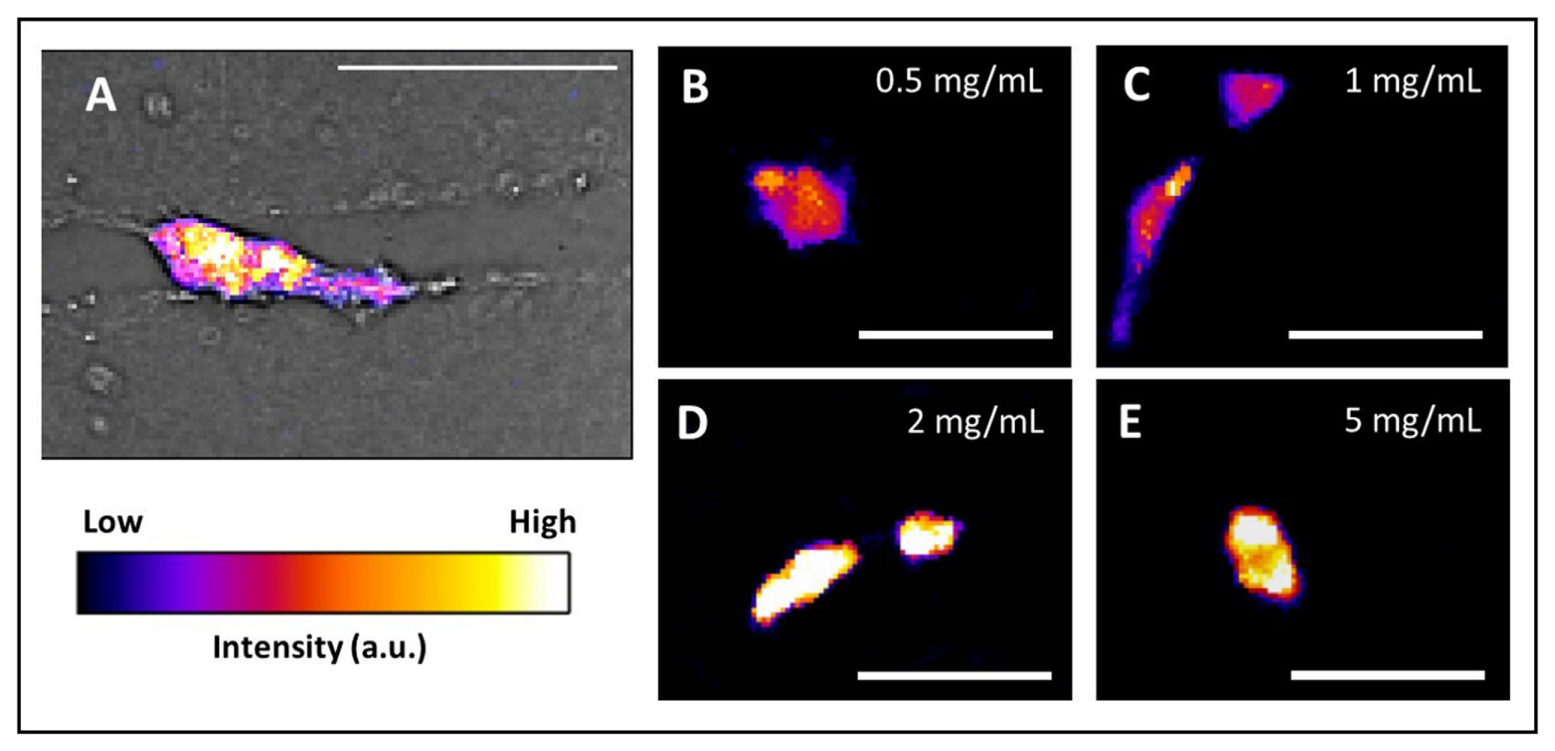
The PercevalHR probe can be used to image ATP:ADP ratios in various microenvironments. (**A**) Heat map of PercevalHR/pHRed probe in MDA-MB-231 cell migrating in a microfabricated collagen microtrack (scale bar = 50 µm). (**B**–**E**) Heat map of PercevalHR/pHRed probe in MDA-MB-231 cells in 3D collagen densities of **B**) 0.5 mg/mL, **C**) 1 mg/mL, **D**) 2 mg/mL, and **E**) 5 mg/mL (scale bar = 50 µm).

By quantifying ATP:ADP in individual live cells, it is possible to probe the role of cell metabolism in migration. The Warburg effect postulates that glycolysis is preferred over mitochondrial respiration in energy production^52^, generating interest in how this altered energy dynamic regulates migration and interaction with the extracellular matrix (ECM)^53^. In dense, more-challenging environments, ATP:ADP ratios in cancer cells are elevated, likely to meet the higher energetic demands of cells navigating the ECM^9^ (Figure 1A). When presented with fewer impediments in aligned matrices, that ratio significantly drops, suggesting that cells can specifically tune energy levels to meet the demands of the surrounding matrix architecture. Additionally, PercevalHR has been utilized to explore the relationship between confinement and bioenergetics during migration, where increased confinement typically imposes a higher energetic demand on cells^30^ (Figure 1B–E). Moreover, cells tend to follow the path of least resistance in these energy requirements, as cells preferentially migrate into less confined, potentially less energy-intensive pathways. The ability to monitor ATP:ADP levels in single cells and correlate those with migration metrics like velocity has been a powerful tool in demonstrating that cells adapt energy levels based on their migration behavior and microenvironment.

In addition to modulating ATP:ADP levels during migration, cells exhibit changes in energy localization in response to mitochondrial positioning that can significantly affect migratory ability^51^. Recent data suggest that intracellular energy distribution, mapped using PercevalHR, modulates migration and controls features such as protrusive and adhesive activity and, consequently, migration speed in embryonic fibroblasts. When ATP:ADP ratios are reduced at the cell periphery and mitochondria become restricted to the perinuclear space, decreased membrane dynamics and adhesion stability mediated by the Rho-GTPase Miro-1 attenuate migration in both single and collective cells. Evidence suggests that Miro-1 is required for migration and wound healing during injury of epithelial cells^54^, highlighting the importance of mitochondrial function in mediating cell migration.

PercevalHR has proven a useful, viable tool not only *in vitro* but also *ex vivo*^50^. PercevalHR-transduced cells were injected into the subventricular zone of adult mice, and brain tissue sections were removed and time-lapse imaged. Cells actively and dynamically changed ATP:ADP ratios, promoting autophagy to regulate the pace of migratory and stationary phases in the cell. This work lays the foundation for both further use of probes in highly relevant, *ex vivo* and *in vivo* environments as well as understanding how energy consumption is essential in key migratory processes. Since so few techniques for investigating cell migration are applicable to *in vivo* studies, molecular probes hold the potential to further elucidate more relevant and useful therapeutic targets for many aggressive diseases.

### Visualizing NADH:NAD+ redox state

The cytosolic NADH:NAD+ redox state is an important indicator of bioenergetics that drives the flow of electrons during the electron transport chain. Nicotinamide adenine dinucleotide (NAD) reduces to NADH when it accepts an electron, and both are essential cofactors in glycolysis, the citric acid cycle, and OXPHOS^55^. The ratiometric Peredox probe reports NADH:NAD+ ratios in live cells by incorporating the bacterial redox-sensing, transcriptional repressor Rex that competitively binds NADH and NAD+ and increases fluorescence upon binding to NADH^56,57^. In addition to correlating the redox state with glycolysis in bacterial cells^58,59^, neural cells^60^, neuroblastoma and epithelial cells^56^, and melanoma cells^61^, Peredox has been used to elucidate the role of key glycolytic and OXPHOS mediators in cancer cell invasion^61^.

Recently, Peredox was used to interrogate the role of Citrin, the mitochondrial transporter, in increasing cancer cell invasion by measuring the relative levels of NAD+ and NADH during energy production in cells where the transporter was silenced^61^. While NAD+ and NADH levels were both found to decrease in cells lacking Citrin, levels of glycolysis and OXPHOS similarly decreased, suggesting that Citrin aids in providing intermediates for both energetic pathways. However, Peredox is not the only probe that takes advantage of the competitive binding of Rex. Similar variations of the construct have been used to highlight glycolysis as a determinant of cell migration in development. A Rex probe paired with yellow fluorescent protein reported that neural crest migration depends heavily on glycolysis and is abrogated in cells utilizing OXPHOS^62^. This has been echoed in recent studies, where increased glycolysis and its intermediates were linked to increased migration^29,63–68^. These probes allow visualization of the inherent metabolic plasticity of cells and how migratory phenotypes are altered when cells utilize different energy pathways.

### 2-NBDG probe: visualizing glucose uptake

Glucose fuels glycolysis, releasing ATP and other factors that feed into OXPHOS. 2-NBDG is a modified, fluorescent glucose analog that is effectively taken up by cells, but not utilized during glycolysis, and thus serves to report glucose uptake in cells^69^. This probe has been used as an indicator of glycolysis to study cancer cell migration in particular, as cancer cells are known to exhibit increased glucose uptake and glycolysis to support increased proliferation. Increased 2-NBDG fluorescence has been correlated with increased migration in cells^62^ and directly related to an increase in ATP hydrolysis^9^. Not only has 2-NBDG been used in *ex vivo* live tissues successfully^70^ but also, in future work, it could be used to track glucose uptake and therefore relative glycolytic rates *ex vivo* and *in vivo* during migration through highly relevant microenvironments to investigate dynamic metabolic plasticity.

### Visualizing H_2_O_2_ gradients

Cells can respond to various environmental cues to regulate and drive migration. The “redox status” of cells, describing the relative levels of regulators of oxygen, can be determined by these cues and is regulated by the production of intermediates such as reactive oxygen species (ROS) like hydrogen peroxide (H_2_O_2_)^71^. H_2_O_2_ serves as an important second messenger since it is an oxidizing agent during cell metabolism^72^. Multiple probes have been developed incorporating a fluorescent protein with the H_2_O_2_-sensing protein OxyR to obtain real-time, live-cell tracking of H_2_O_2 _localization in the cell to gain insight on the redox state and H_2_O_2_ gradient^72–74^. The recently developed and most sensitive of these probes is HyPer 7, which can detect very low concentrations of H_2_O_2_ while being pH stable^72^. Using this probe, protrusion formation and cell polarization was shown to rely significantly on the H_2_O_2_ concentration at the leading edge of cells and the steepness of the H_2_O_2_ gradient from the protrusion to the cell body was found to directly correlate with the stability of the protrusion. This gradient, observed using a HyPer variant, has additionally been reported to change based on mechanical environmental cues, for instance confinement, to alter lamellipodia formation and induce migration toward available oxygen^75^. Though further work is required to understand exactly how this H_2_O_2_ gradient and signaling directs migration and cell polarization, the increased sensitivity of this probe, and others, has provided a unique avenue for further exploration.

While these probes correlate migratory events with real-time metabolic readouts, they also point to mechanisms by which cell migration is directly affected by intracellular bioenergetics. In the future, the use of these probes *in vivo* could aid in unveiling migratory mechanisms of cells experiencing altered biochemical and mechanical cues that are often too complex to replicate *in vitro*.

## Collective migration and leader/follower heterogeneities

### Metabolism in collective migration and 3D culture models

**Figure 2.**
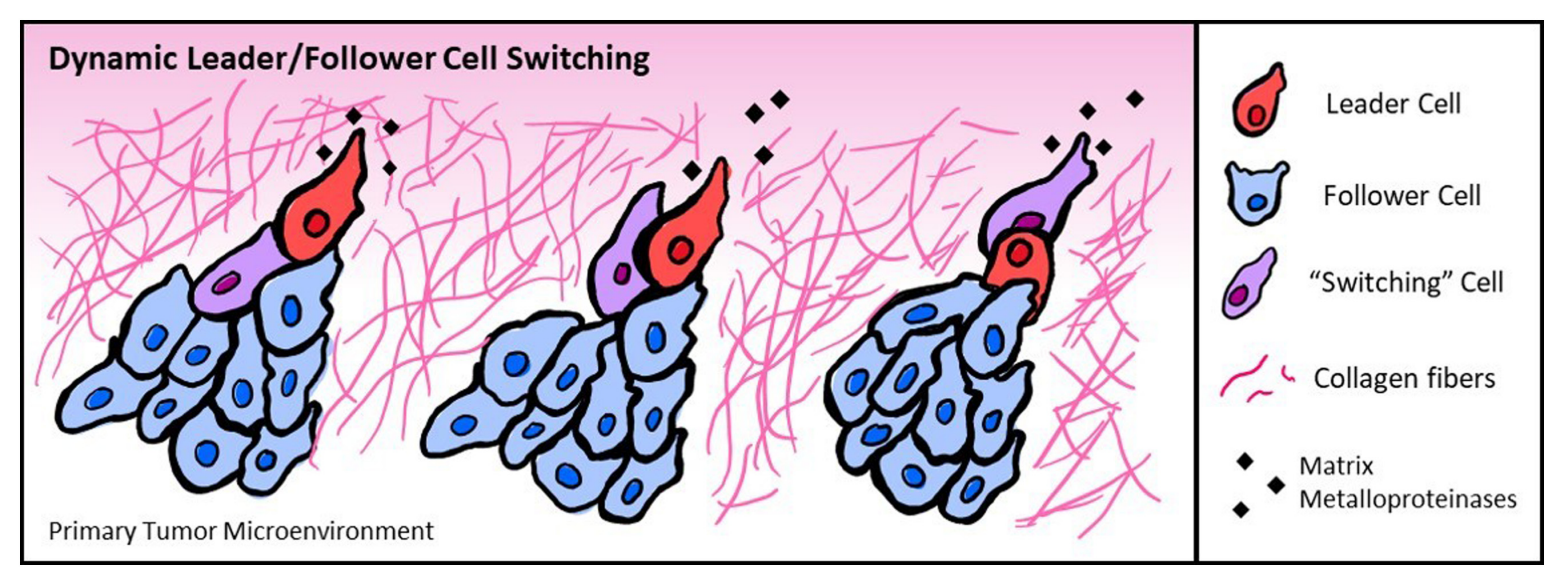
Dynamic leader/follower switching during collective migration allows energy-depleted leader cells to be replaced with new leader cells at collective cell front. Cues from the microenvironment such as collagen density may alter energy utilization and migratory behavior. Matrix metalloproteinases, potentially activated because of acidification of the surrounding microenvironment during glycolysis, can help to degrade the extracellular matrix.

Recently, metabolic heterogeneities in collective cell populations have gained increased interest. Collective cell migration, in contrast to single cell migration, relies not only on cues from the microenvironment but also on signaling and interaction with surrounding cells. Cells migrating *en masse* can show differential responses to surrounding cues^76^ and are thought to minimize energetic costs compared to single cells, effectively preserving energy for cells to navigate more challenging environments^9,77–79^. One could speculate that collectively migrating cells utilize different strategies to achieve cell–cell communication and collective invasion, as cells may experience metabolic rewiring and/or swap positions due to leader cell energy depletion to efficiently invade^31,80^ (Figure 2). Glycolytic regulation of ATP/ADP ratios in cells has also been reported to play a key role in cytoskeletal remodeling, cell migration, and leader cell competitiveness during events like vessel sprouting^81^. Thus, ATP generation and trafficking in the cell is essential to a variety of collective processes.

Metabolic plasticity in collective migration may allow cells to meet dynamically changing energy needs within a heterogeneous microenvironment that presents cells with varying obstacles. For example, when cells encounter denser microenvironments and energy demands are increased, ATP has been shown to be generated through the OXPHOS pathway via mitochondrial trafficking to the leading edge of individually migrating cells, opposing the traditional Warburg theory^8^. In these high-density, energy-demanding environments, cells typically switch from single cell to collective migration^78^, suggesting that collective migration may provide some protection from energy depletion and migratory advantage. Similarly, increased substrate stiffness has been shown to result in higher collective cell migration speed, persistence, and area of multicellular protrusions^82,83^. Because stiffness gradients within the ECM can also affect the directionality and coordination of collective cell movements^84,85^, it is likely that varying stiffnesses result in an altered metabolic response in collective cells. As cell clusters may simultaneously experience heterogeneous stiffness and stress distributions due to the organization of the microenvironment^85^, determining how cells meet these localized energy demands and employ different energy production strategies is integral in probing collective cell behavior.

As increasing interest in collective cell energetics emerges, 3D culture models have gained attention as tools for investigating mechanisms of collective cell migration in relation to disease progression, especially in tumor metastasis^86–92^. Among the various collective migration models available, spheroids and organoids have been specifically used to investigate the relationship between collective cell migratory ability and environmental cues^93–97^. These highly relevant tools have been used in attempting to unveil potential mechanisms for how cell heterogeneity, migratory modes, and the microenvironment all contribute to a dynamically changing metabolic profile of migrating cells^31,32,80,98–102^.

Environmental cues like peroxide gradients or hypoxia can be formed in 3D culture models that can be used to specifically probe leader/follower dynamics in the context of the cellular redox state. When human apurinic/apyrimidinic endonuclease-1 (APE1), a key regulator of ROS production and redox state, was inhibited in breast cancer cells, collective migration was significantly inhibited, suggesting that ROS generation and regulation plays an important role not only during single cell migration but also in collective invasion and migration^103^. Given these data, it is possible that the redox status of individual cells during collective cell migration contributes to the metabolic differences between leader and follower cell populations and using HyPer and other peroxide probes may provide more information when used in a collective context. At high doses, H_2_O_2_ treatment has been shown to modulate junction proteins in collective cells and effectively inhibit migration in various cancer cell lines^104–106^. Additionally, hypoxia gradients have been shown to induce collective-to-amoeboid transition in cancer cells, in which single cells in collective masses are able to escape the leading edge and switch to an amoeboid migratory mode^107^. This suggests that even within collective masses, cells react on an individual level to changing hypoxic gradients and ROS modulation to more efficiently navigate their external environment. Future use of peroxide and ROS-related probes in models like spheroids or organoids should reveal how leader/follower cells may rely on various oxygen levels to fill their roles during collective migration.

### Leader/follower dynamics

Observing the metabolic plasticity of collectively migrating cells has led to a recent focus on the distinction between the independent roles of leader/follower cells. The formation of phenotypically unique leader cells from follower cells is reportedly dependent on dynamic collective stresses in follower cells that can in some instances pull leader cells into position^108^. As energy utilization in this mechanism is still not fully understood, molecular probes and fluorescent labeling have been employed to determine how the differences between these two distinct cell groups are established and affected by metabolic requirements. For instance, cells expressing GFP-labeled keratin-14 in primary breast tumor organoids were found not only at the leading edge of collective cells to direct migration but also in follower positions that can engage in leader/follower switching events in response to both microenvironmental cues and intracellular energy needs^31,109,110^. Utilizing RFP-tagged and CycleTrak-labeled MDA-MB-231s, it has been shown that cells guided by environmental cues may compete for a dynamically changing leader cell position to minimize energetic costs during ECM invasion^31,77^. Thus, probing individual cell behavior with various fluorescent biomarkers has further clarified the metabolic determinants of different subpopulations during collective migration; moreover, these findings have suggested that the regulation of leader/follower cell metabolic profile by the surrounding microenvironment is critical to collective migration.

### Metabolic and phenotypic heterogeneities in leader/follower cells

Molecular probes are a powerful tool for dissecting the processes of collective migration, especially in the context of bioenergetics and cancer. Unlike Seahorse, which requires large cell populations^29,51,111^, molecular probes allow for metabolic profiling of individual cells in collective migration, highlighting the spatial and temporal heterogeneities in leader/follower dynamics. The PercevalHR probe has been used to observe available energy in leader cells during collective cancer cell invasion *in vitro* through dense collagen, which is directly related to leader cell lifetime and leader/follower positions^31^. Using the 2-NBDG probe, it has been separately reported that leader cells exhibited higher glucose uptake than follower cells, suggesting increased glycolysis^31^, while another reported the preferential usage of OXPHOS by leader cells^32^. Though it is clear that collective cell populations maintain complex metabolic heterogeneity, how cells utilize different metabolic pathways during collective migration remains unclear. These real-time cell tracking techniques not only shed light on cell metabolism during migration but also, more importantly, point to the need for further investigation into leader versus follower cell behavior.

To understand the needs of individual leader and follower cells during migration, researchers have taken advantage of the ability to physically sort heterogeneous subpopulations based on their distinct phenotypic, genotypic, and metabolic profiles using tools such as photoactivation and spatiotemporal genomic and cellular analysis (SaGA)^31,112^. These techniques employ the use of photoconvertible tags to identify and mark individual cells in collective populations that can then be sorted and expanded into discrete subpopulations. By creating separate populations characterized by mutations affecting invasive capability^113^, highly invasive leader cells were shown to rely on focal adhesion kinase–fibronectin signaling to promote invasion, while follower cells instead are recruited to leader cells via irregular VEGF-based vasculogenic signaling^112^. Further definition of unique subpopulations can also be determined using single-cell sequencing, probing the metabolic heterogeneities in bulk expression profiles to link metabolic gene expression during *in vitro* cell migration with *in vivo* metastasis^114–117^. By specifically studying these populations, a fuller understanding of their interactions and roles in collective cell migration can be gained, particularly in the context of invasive diseases like cancer.

Recently, it has been shown that cells can be sorted without the use of specific probes or photoactivation but rather based on autofluorescence of the metabolic intermediates NAD(P)H and FAD^66,118–120^. High levels of NAD(P)H autofluorescence may serve as a useful biomarker for increased OXPHOS^119^, though it may be difficult to fully parse the contributions of NADH and NADPH to metabolism and whether these changes specifically point to mitochondrial respiration or glycolysis^57^. Nonetheless, autofluorescence has provided a probe-free avenue for exploring the metabolic preference in cells that could lend to easier characterization of migratory metabolism in the future.

As studies continue to investigate the metabolic activity of leader/follower cell populations, conflicting reports have emerged regarding the utilization of glycolysis and/or mitochondrial respiration during collective migration. In cancer, leader cells have been shown in some instances to rely on glycolysis, as demonstrated by increased ATP/ADP ratios and increased glucose uptake^31^, while others show a dependence on OXPHOS^32^, as shown by increased sensitivity of leader cells to mitochondrial-targeting treatment. During glycolysis, the tumor microenvironment is acidified owing to the increase in lactate production by cells^52,121^, which has been linked to the activation of matrix metalloproteinases^122,123^, important mediators of matrix degradation. This evidence supports the idea that leader cells rely more heavily on glycolysis, potentially to aid in matrix remodeling for migration, and further highlights how leader/follower phenotypic heterogeneity may be sustained or partially altered by the microenvironment. As future studies continue to reveal distinct leader/follower profiles, these opposing reports may be resolved with further investigation into how leader/follower dynamics and complex microenvironmental cues regulate migratory metabolism.

## Conclusions

In very recent work, cell migration research has focused on understanding the role of metabolic heterogeneities in cell migration. Studies have narrowed from visualizing intracellular energetics in single cell and collective groups of cells to sorting cells into specific populations and characterizing the phenotypic and genotypic differences in these subpopulations. This is made feasible through the use of photoactivation and cell sorting, with characterization made simple using molecular probes that allow visual and quantitative comparison of metabolic processes in migrating cells. However, tools and techniques for monitoring and quantifying energy production and consumption in real time *in vivo* are still lacking and will be essential in understanding cellular energy status of migrating cells in highly complex and relevant environments. Additionally, in primary or patient-derived cell lines where cells are more fragile and last shorter time periods *in vitro*, molecular probes requiring several passages for transduction and even selection can be technically difficult to utilize. Therefore, the development of a less-invasive tool to visualize and quantify intracellular energy status in these cells will be particularly useful in future cell migration work.

Recent research has revealed that individual cell populations hold key heterogeneities that independently contribute to the migration of collective cell groups as a whole, with metabolic intermediates playing an essential role in determining the function and role of these cells. Whether these heterogeneities are inherent in the cell or dependent on surrounding conditions has yet to be fully explored and is of key interest in future work. As it is still unclear which metabolic pathways are prioritized by specific cell subpopulations, further investigation into these questions is essential to fully understand the mechanisms of both single and collective cell migration, especially in the context of a complicated disease-developing environment.
